# Interleukin-21 promotes thymopoiesis recovery following hematopoietic stem cell transplantation

**DOI:** 10.1186/s13045-017-0490-3

**Published:** 2017-06-14

**Authors:** Aurélie Tormo, Fatemeh Khodayarian, Yun Cui, Edouard Al-Chami, Reem Kanjarawi, Beatriz Noé, Huijie Wang, Moutih Rafei

**Affiliations:** 1 The Department of Pharmacology and Physiology, Université de Montréal, 2900 Edouard-Montpetit BLVD, Montréal, Québec H3T 1J4 Canada; 2 The Department of Microbiology, Infectious Diseases and Immunology, Université de Montréal, 2900 Edouard-Montpetit BLVD, Montréal, Québec H3T 1J4 Canada; 3 The Department of Microbiology and Immunology, McGill University, 3775 University Street, Montréal, Québec H3A 2B4 Canada

**Keywords:** IL-21, Hematopoietic stem cells, Thymopoiesis, Spleen, Graft-versus-host disease, Regulatory B cells, Graft-versus-tumor, Humanized mice

## Abstract

**Background:**

Impaired T cell reconstitution remains a major deterrent in the field of bone marrow (BM) transplantation (BMT) due to pre-conditioning-induced damages inflicted to the thymi of recipient hosts. Given the previously reported thymo-stimulatory property of interleukin (IL)-21, we reasoned that its use post-BMT could have a profound effect on de novo T cell development.

**Methods:**

To evaluate the effect of IL-21 on de novo T cell development in vivo, BM derived from RAG2p-GFP mice was transplanted into LP/J mice. Lymphocyte reconstitution was first assessed using a hematological analyzer and a flow cytometer on collected blood samples. Detailed flow cytometry analysis was then performed on the BM, thymus, and spleen of transplanted animals. Finally, the effect of human IL-21 on thymopoiesis was validated in humanized mice.

**Results:**

Using a major histocompatibility complex (MHC)-matched allogeneic BMT model, we found that IL-21 administration improves immune reconstitution by triggering the proliferation of BM Lin^−^Sca1^+^c-kit^+^ (LSK) subsets. The pharmacological effect of IL-21 also culminates in the recovery of both hematopoietic (thymocytes) and non-hematopoietic (stromal) cells within the thymi of IL-21-treated recipient animals. Although T cells derived from all transplanted groups proliferate, secrete various cytokines, and express granzyme B similarly in response to T cell receptor (TCR) stimulation, full regeneration of peripheral naïve CD4^+^ and CD8^+^ T cells and normal TCRvβ distribution could only be detected in IL-21-treated recipient mice. Astonishingly, none of the recipient mice who underwent IL-21 treatment developed graft-versus-host disease (GVHD) in the MHC-matched allogeneic setting while the graft-versus-tumor (GVT) effect was strongly retained. Inhibition of GVHD onset could also be attributed to the enhanced generation of regulatory B cells (B10) observed in the IL-21, but not PBS, recipient mice. We also tested the thymopoiesis-stimulating property of human IL-21 in NSG mice transplanted with cord blood (CB) and found significant improvement in de novo human CD3^+^ T cell development.

**Conclusions:**

In sum, our study indicates that IL-21 represents a new class of unforeseen thymopoietin capable of restoring thymic function following BMT.

**Electronic supplementary material:**

The online version of this article (doi:10.1186/s13045-017-0490-3) contains supplementary material, which is available to authorized users.

## Background

BMT is commonly used in the clinic as a curative therapy for a variety of life-threatening diseases of lympho-hematopoietic origin [1–7]. Prior to BMT, patients undergo myeloablative or non-myeloablative conditioning regimens consisting of radiation, chemotherapy, and/or administration of immunosuppressive drugs [1–7]. Although innate immunity mediated by myeloid and natural killer cells recovers relatively quickly in patients of all ages, T cell reconstitution requires years and even then rarely re-establishes a fully competent T cell repertoire [8–10]. This prolonged post-transplant lymphoid deficiency is associated with increased risks of (i) cancer relapse or development of secondary malignancies [11–17], (ii) infection [18–21], and (iii) reduced responses to immunotherapies such as vaccination [5, 22]. In line with this notion, a clinical study based on 42 mantle cell lymphoma patients who underwent BMT revealed that absolute lymphocyte count recovery is a powerful prognostic factor for clinical outcome [23]. Furthermore, accelerated lymphocyte recovery culminates in improved responses to immune-based treatments as previously achieved with the combined use of rituximab and BMT [23–26]. Thus, identifying factors capable of inducing de novo intrathymic T cell development (thymopoiesis) in patients undergoing BMT remains a central goal in the field.

IL-21 is the most recently identified member of the common γ-chain family of cytokines [27]. Some of its pleiotropic functions include (i) promoting Th17 differentiation in the presence of transforming growth factor-β [28], (ii) activating NK and CD8 lymphocytes [29, 30], (iii) desensitizing responder cells to the inhibitory effects of regulatory T cells (Tregs) [31], and (iv) acting as a switch for IgG production in B cells [32]. Although not required for hematopoiesis, in vivo expansion of bone marrow (BM) progenitors was observed in response to IL-21 overexpression [33]. Consistently, Simard et al. showed that IL-21 signaling in BM-derived B cell progenitors accelerates the development of immature/mature B cells by regulating the expression of Blimp1, Aid, and the germline transcript gamma 2b [34]. For the T cell lineage, however, IL-21 is not necessary for thymocyte differentiation due to normal thymopoiesis in mice exhibiting an IL-21 receptor (IL-21R) deficiency [27, 32]. Paradoxically, we previously reported that TCR engagement on double-positive (DP) thymocytes triggers the de novo expression of IL-21R [35]. Although DP thymocytes undergo a three- to fourfold expansion in response to IL-21 treatment in vitro, no differentiation towards CD8^+^ single-positive (SP) T cells could be detected [35]. As no mitogen has ever been described for DP thymocytes, we then tested the thymopoiesis-stimulating ability of IL-21 in models of acute and chronic thymic atrophy. For instance, IL-21 administration to mice suffering from corticosteroid-induced thymic atrophy led to double-negative (DN) and DP thymocyte expansion, which resulted in accelerated recovery of thymic function [36]. In wild-type (WT) aged mice displaying naturally induced thymic atrophy, IL-21 enhanced the output of recent thymic emigrants (RTEs), which qualitatively changed the peripheral T cell pool [37]. On top of correcting several T cell defects commonly seen with aging such as impaired proliferation and biased cytokine production, an enhanced anti-tumoral response to a Trp2-based melanoma cancer vaccine was achieved in aged mice pre-conditioned with IL-21 [37]. The overall significance of these findings epitomizes IL-21 as a potent pharmacological agent for stimulating thymopoiesis and serves as the basis to further investigate its use in T cell reconstitution following allogeneic BMT.

## Methods

### Cell line and mice

The P815 cell line was purchased from ATCC (Manassas, VA, USA). The RAG2p-GFP transgenic mice were kindly provided by Dr. M. Nussenzweig (Rockefeller University, NY, USA). C57BL/6 (H2-K^b^), IL-7^−/−^ (C57BL/6 background, H2-K^b^), IL-21R^−/−^ (C57BL/6 background, H2-K^b^), LP/J (H2-K^bc^), BALB/c (H2-K^d^), MμMT (H2-K^b^), and NSG mice were purchased from the Jackson Laboratory (Bar Harbor, ME). Littermate mice were interbred and housed in a pathogen-free environment at the animal facility of the Institute for Research in Immunology and Cancer. Animal protocols were approved by the Animal Care Committee of Université de Montréal.

### Antibodies, cytokines, reagents, and kits

Recombinant IL-7 (#217-17) and IL-21 (#210-21 for mouse and #200-21 for human) cytokines were purchased from PeproTech (Rocky Hill, NJ, USA). The flow cytometry antibodies CD3 (17A2), CD4 (GK1.5), CD5 (53-7.3), CD8 (53-6.7), NK1.1 (PK136), CD19 (1D3), CD25 (PC61), CD34 (RAM34), CD44 (1M7), CD45 (30-F11), CD62L (MEL-14), CD135 (A2F10.1), IL-10 (JES5-16E3), human CD3 (UCHT1), human CD4 (RPA-T4), human CD8 (SK1), human CD13 (WM15), human CD45 (H130), and Cytofix/Cytoperm Kits were purchased from BD Pharmingen (San Diego, CA, USA). FOXP3 (FJK-16S), granzyme B (NGZB), and human CD161 (HP-3G10) antibodies as well as monensin were purchased from eBioscence/Thermo Fisher (Waltham, MA, USA). Quantikines were purchased from R&D Systems (Minneapolis, MN). CD3-CD28 beads, CellTrace^TM^, and TRIzol were purchased from Invitrogen (Burlington, ON, CA). Lipopolysaccharide (LPS), PMA, and ionomycin were purchased from Sigma (St. Louis, MO, USA). All cell isolation kits were purchased from STEMCELL Technologies (Vancouver, BC, Canada). RNA extraction kits were purchased from Qiagen (Toronto, ON, Canada).

### BMT and immune recovery assessment

Irradiation of LP/J (11 Gy), IL-7^−/−^ (8.5 Gy), or BALB/c (8.5 Gy) recipient female was conducted prior to transplantation with T cell-depleted 5 × 10^6^ RAG2p-GFP-derived BM cells by intravenous injection. On the following day, mice were administered IL-21, IL-7 (both at 50 μg/kg), or equivalent volume (100 μl) of sterile PBS by intraperitoneal injections every 2 days over a period of 2 weeks (total of 6 injections). Immune reconstitution was assessed weekly using both the Scil vet ABC Plus^+^ hematological analyzer and flow cytometry on collected blood samples. BM, thymic, and spleen analyses were conducted as previously described [37].

### GVHD/GVT induction and analysis

To induce GVHD, T cell-depleted BM derived from RAG2p-GFP or MμMT mice was supplemented with 10^6^ enriched T cells (from RAG2p-GFP), then used to transplant irradiated LP/J recipient animals followed by PBS/IL-21 treatments as previously described. For assessment of GVHD severity, mice were scored individually for five clinical parameters on a scale from 0 to 2 for weight loss, posture, activity, fur, and skin lesions. For GVT experiments, transplanted LP/J mice where challenged intravenously with 5 × 10^5^ P815 tumor cells the day following BMT. Mice were sacrificed when weight loss reached ≥20% and/or the sum of total clinical scores was above 8.

### Proliferation, cytokine measurements, and intracellular staining

T cells were isolated from splenocyte suspension by negative selection and were stimulated with CD3-CD28 dynabeads. Cells or supernatants were collected 48 h post-stimulation for further analyses. For intracellular detection of cytokines, stimulated T cells were stained for CD4/CD8 prior to intracellular staining according to the manufacturer’s instructions. FOXP3 staining on T cells and IL-10 analysis in B cells were conducted as previously described [37, 38].

### Expression analysis of transcription factors

T cells from spleens of transplanted mice were sorted in 900 μl TRIzol (10^6^ cells per tube) followed by RNA extraction. Reverse transcription was performed by qPCR. Target gene values were normalized to endogenous control *Gapdh*.

### NSG transplantation using CB

Cord blood (CB) units were obtained from the Ste-Justine blood bank (Montreal, Qc, Canada) following ethics approval. Mononucleated cells were first isolated by density centrifugation, then depleted from human T cells prior to their transplantation (10^5^ cells) in sublethally irradiated (3 Gy) NSG mice [39–41]. Transplanted NSG mice were then intraperitoneally injected with human IL-21 (50 or 100 μg/kg) or human IL-7 (50 μg/kg) versus equivalent volumes of PBS (100 μl) every 2 days over a period of 2 weeks (total of 6 injections). Peripheral blood was collected every 2 weeks and analyzed as described above. Transplanted mice were sacrificed at the end of the experiment to analyze their thymi and spleens.

### Statistical analyses

*P* values were calculated using the ANOVA and log-rank statistical test where applicable.

## Results

### IL-21 administration post-BMT correlates with accelerated lymphoid recovery

**Fig. 1:**
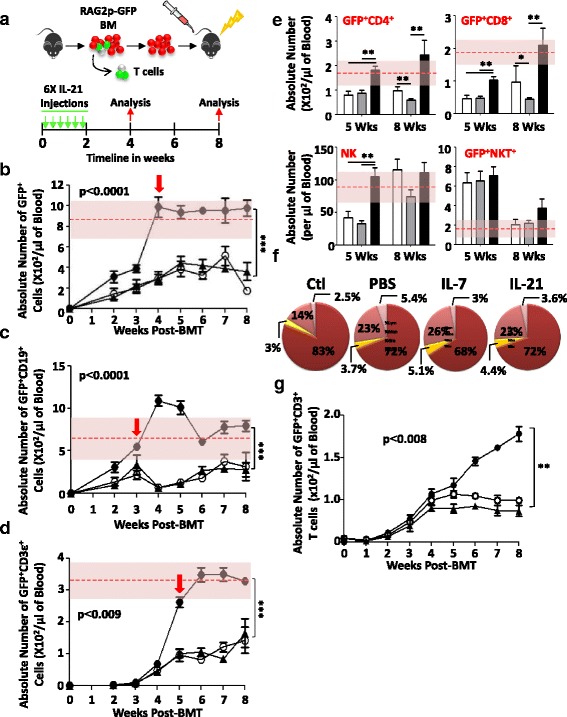
Peripheral T cell reconstitution. **a** Schematic cartoon summarizing the BMT protocol. Absolute counts of GFP^+^ (**b**), GFP^+^CD19^+^ (**c**), or GFP^+^CD3^+^ (**d**) T cells in peripheral blood of transplanted mice (*n* = 10/group). The *red dashed line* within the *pink-shaded area* represents the mean physiological level of the GFP^+^ population of interest being analyzed +1 standard deviation calculated using blood samples collected from 10 unirradiated RAG2p-GFP mice. The treatment groups are represented as follows: PBS (*empty circle*), IL-7 (*filled triangle*), and IL-21 (*filled circle*). **e** Absolute counts of GFP^+^CD4^+^, GFP^+^CD8^+^, NK^+^, or GFP^+^NKT^+^ cells at 5 and 8 weeks post-BMT (*n* = 10/group). The PBS group is represented by *white bars*, IL-7 by *gray bars*, and IL-21 by *black bars*. **f** Pie charts reflecting the percentages of all major blood-circulating immune populations. The represented groups are follows: lymphocytes in *dark red* (68–83%), granulocytes in *pink* (14–26%), monocytes in *yellow* (3–5.1%), and eosinophils in *light pink* (2.5–5.5%). Control mice (*Ctl*) represent unirradiated mice. **g** A representative BMT experiment conducted in IL-7^−/−^ (on a C57BL/6 background) recipient mice (*n* = 10/group). The treatment groups are represented as follows: PBS for RAG2p-GFP-derived BM → IL-7^−/−^ (*empty circle*), IL-21 (50 μg/kg) for RAG2p-GFP-derived BM → IL-7^−/−^ (*filled triangle*), and a technical control consisting of PBS administration to RAG2p-GFP-derived BM → WT C57BL/6 setting (*filled circle*). All experiments were conducted at least three times with *P < 0.05, **P < 0.01, and ***P < 0.0001

Amongst all immune subsets, T cells are the slowest to emerge (if any) post-BMT [8–10]. Following their development in the thymus, newly generated CD4^+^ and CD8^+^ SP thymocytes, which are referred to as RTEs, egress to the periphery where they continue their maturation in secondary lymphoid organs [42]. To detect RTEs, peripheral blood samples are usually used to quantify TCR excision circle content, which represents a direct reflection of TCR rearrangements during intrathymic T cell development [43–45]. However, TCR excision circle quantification by PCR has several limitations and remains an indirect method that needs to be interpreted with caution [43–45]. Alternatively, RTEs and newly generated B cells both can be monitored using RAG2p-GFP mice, where GFP expression is controlled by the *Rag2* promoter activity during T and B lymphopoiesis [37, 46, 47]. Therefore, we first quantified the physiological ranges of total GFP^+^ (RTEs and newly developed B cells), GFP^+^CD19^+^ (newly developed B cells), or GFP^+^CD3^+^ (RTEs) cells in circulation using blood samples collected from unirradiated control RAG2p-GFP mice prior to conducting BMT experiments (Fig. 1a). According to these ranges (displayed as pink-shaded areas), IL-21 administration to LP/J mice accelerated significantly lymphoid recovery in contrast to PBS- or IL-7-treated mice (Fig. 1b). In particular, GFP^+^CD19^+^ cells (Fig. 1c) reached physiological levels 3 weeks post-BMT whereas GFP^+^CD3^+^ levels (Fig. 1d) were normalized by the 5th week (indicated by red arrows) following transplantation. Upon further dissection, enhanced re-establishment of peripheral GFP^+^CD4^+^, GFP^+^CD8^+^, GFP^+^NKT^+^, and NK^+^ cells were observed at both 5th and 8th weeks post-BMT in IL-21-treated mice (Fig. 1e) with no overrepresentation of myeloid versus lymphoid cells in any of the transplanted groups (Fig. 1f).

Given the profound effect of IL-21 on T cell recovery post-BMT, we next examined whether it displays redundant function with IL-7, the major thymopoietin known to support T cell development [48]. To test this hypothesis, peripheral GFP^+^CD3^+^ T cells were quantified in IL-7^−/−^ recipient mice transplanted with T cell-depleted RAG2p-GFP BM. Although no differences in the generation of GFP^+^CD3^+^ T cells were depicted between PBS- and IL-21-treated IL-7^−/−^ recipient animals, the overall RTE levels were significantly lower compared to WT C57BL/6 recipient mice on the PBS regimen (Fig. 1g). These results clearly indicate that IL-21 can indeed accelerate T cell recovery but requires endogenous IL-7 signaling to exert its effects.

### IL-21 triggers the expansion of BM progenitors

**Fig. 2:**
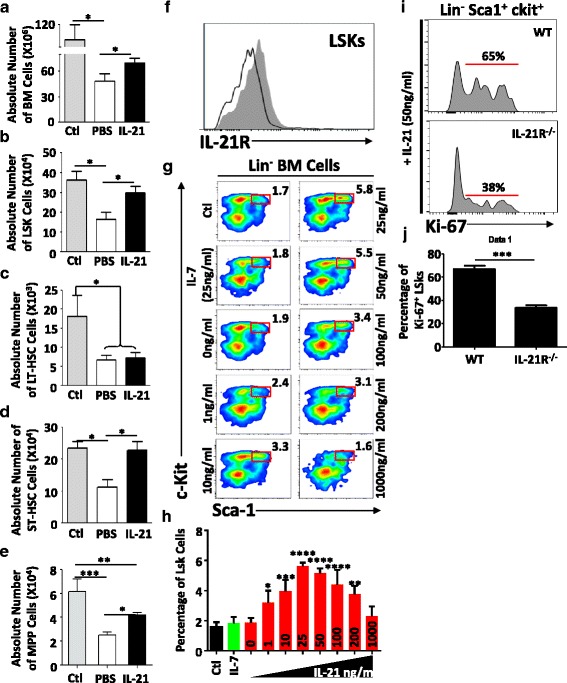
BM engraftment. Absolute counts of **a** total BM cells, **b** LSKs, **c** LT-HSCs, **d** ST-HSCs, and **e** MPPs. Unirradiated mice were used as controls. **f** Representative flow cytometry analysis of IL-21R expression on the surface of LSKs. Isotype control is shown in *black line* whereas IL-21R staining is shown in *filled gray histogram*. **g** Representative flow cytometry experiment analyzing the frequency of LSKs in response to ascending doses of IL-21 (0–1000 ng/ml). Equivalent volume of PBS or IL-7 at 25 ng/ml was used as controls. **h** Quantification of LSK frequency derived from the experiment shown in **g i** Representative intracellular Ki-67 staining in WT versus IL-21R^−/−^ LSK 48 h post-treatment with 50 ng/ml of IL-21. **j** Quantification of KI-67^+^ LSKs derived from the experiment shown in **i**. All experiments were conducted at least three times with *P < 0.05, **P < 0.01, ***P < 0.0001, and ****P < 0.00001

The thymus lacks self-renewing stem cells [49, 50]. Its function relies therefore on sustained seeding by BM-derived progenitors [49, 50]. Although the nature/phenotype of the thymus-settling population(s) remains a matter of debate, BM progenitors are still considered as the limiting substrate for immune reconstitution post-BMT [49–58]. To determine whether the improved peripheral T cell reconstitution previously observed in LP/J recipient mice was due to a direct effect mediated by IL-21 on the BM compartment, hematopoietic stem cells (HSCs) were analyzed in recipient mice 4 weeks following transplantation using well-defined cell surface markers [59]. Aside from the significant increase in total BM cell number (Fig. 2a), Lin^−^Sca1^+^c-kit^+^ (LSK) counts in IL-21-treated animals were highly comparable to those in unirradiated control mice (Fig. 2b). This observed increase in LSKs was not due to long-term (LT)-HSC expansion (Fig. 2c), but rather to augmented numbers of short-term (ST)-HSCs (Fig. 2d) and multipotent progenitors (MPPs) (Fig. 2e). In fact, the effect of IL-21 on LSKs is not surprising as these BM subsets express the IL-21R (Fig. 2f). To further support our in vivo observations, we supplemented freshly plated BM cells with ascending doses of IL-21 (0–1000 ng/ml) and noticed a dose-depending increase in LSK frequency with an optimal effect reached at 25–50 ng/ml (Fig. 2g, h). Further validations by intracellular Ki-67 staining conducted on WT- versus IL-21R^−/−^-derived BM treated with IL-21 (50 ng/ml) revealed a twofold increase in LSK expansion over basal levels (Additional file 1: Figure S1; Fig. 2i, j). The sum of these results clearly links accelerated lymphoid recovery mediated by IL-21 to the proliferation of BM-resident ST-HSCs and MPPs, which represent the hub of thymus-settling progenitors [49, 51–55].

### Thymopoiesis stimulation by IL-21 re-establishes a peripheral naïve T cell pool

**Fig. 3:**
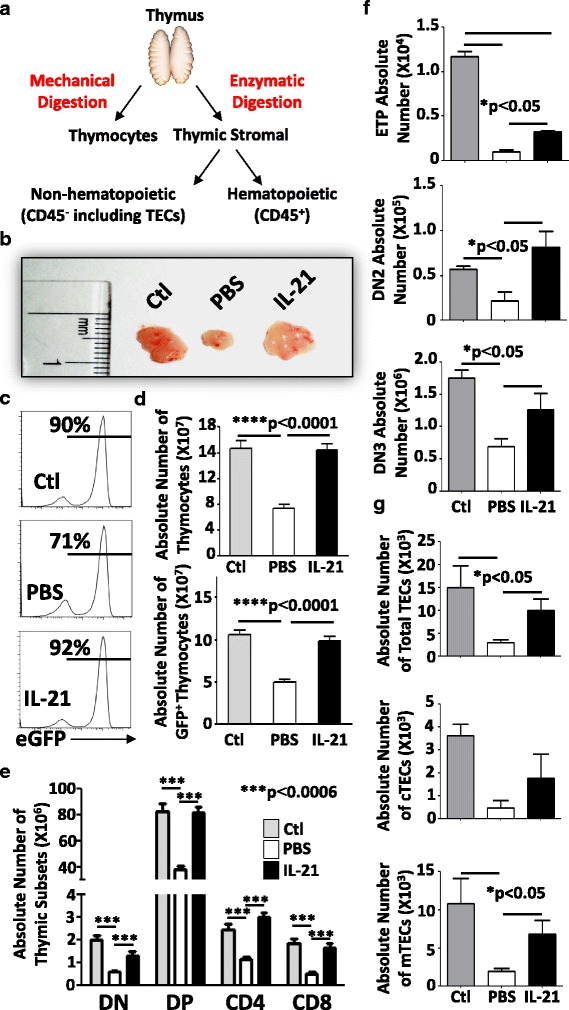
Analysis of thymic tissues. **a** Schematic diagram displaying the approach used for thymic analysis. **b** A representative photograph of thymi derived from treated mice. **c** Representative flow cytometry assessing the GFP frequency of treated vs. ctl mice. **d** Absolute numbers of total (*top*) or GFP^+^ (*bottom*) thymocytes. **e** Absolute counts of all thymic subsets. **f** Absolute counts of ETP (*top panel*), DN2 (*middle panel*), and DN3 (*bottom panel*) thymocytes. **g** Absolute counts of total (*top panel*), cortical (*middle panel*), and medullary (*bottom panel*) TECs. All shown experiments were conducted at least three times with *P < 0.05 and ***P < 0.0001 and *n* = 10/group

The enhanced recovery of ST-HSCs and MPPs post-BMT is indicative of improved engraftment and certainly accounts for the increased peripheral T cell development observed in IL-21-treated animals [60–62]. However, IL-21R was also reported to be expressed on thymic progenitors at the DN and DP stages [35, 36]. This suggests that IL-21 can directly mediate thymocyte proliferation in situ. To confirm this assumption, thymic tissues were resected from transplanted mice 4 weeks post-BMT and analyzed for both the hematopoietic (CD45^+^) and non-hematopoietic (CD45^−^) compartments (Fig. 3a). Interestingly, thymi derived from the IL-21-treated group were comparable in size to those from unirradiated control mice as opposed to PBS-treated animals, which remained atrophied (Fig. 3b). Thymocyte analysis by flow cytometry clearly shows normal GFP frequency levels in IL-21-treated animals (Fig. 3c) with full recovery of both total and GFP^+^ thymocytes (Fig. 3d). All major thymic subsets (Fig. 3e) including early thymic progenitors (ETPs; Lin^−^CD8α^−^CD25^−^c-kit^+^), DN2 (lin^−^CD4^−^CD8^−^CD25^+^CD44^+^), and DN3 (lin^−^CD4^−^CD8^−^CD25^+^CD44^−^) thymocytes (Fig. 3f) recovered in response to IL-21 treatment. Finally, analysis of enzymatically digested thymic tissues revealed significant improvement in total (CD45^−^EpCam^+^), cortical (CD45^−^EpCam^+^UEA1^−^Ly51^+^), and medullary (CD45^−^EpCam^+^UEA1^+^Ly51^−^) thymic epithelial cells (TECs) in the IL-21-treated group when compared to PBS control mice (Fig. 3g).

**Fig. 4:**
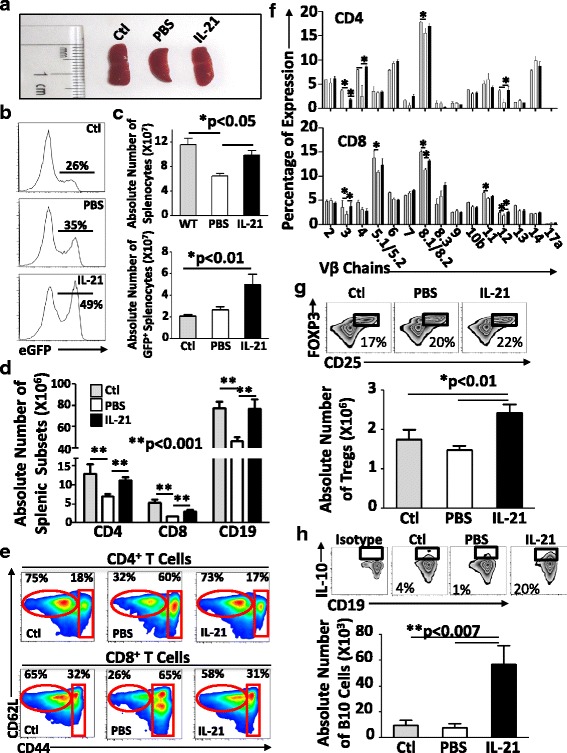
Spleen analysis. **a** A representative photograph of spleens derived from treated mice. **b** Representative flow cytometry of GFP expression profile in splenocytes. **c** Absolute counts of total (*top panel*) or GFP^+^ (*bottom panel*) splenocytes. **d** Absolute counts of all splenic subsets. **e** Representative flow cytometry analysis of naïve (CD62L^+^CD44^−^) and effector/memory (CD62L^+^CD44^+^) CD4^+^ and CD8^+^ T cells. **f** TCRvβ distribution assessed by flow cytometry. **g** Analysis of the frequency (*top panel*) and absolute count of Tregs. **h** Analysis of the frequency (*top panel*) and absolute count of IL-10-producing regulatory CD1d^hi^CD5^+^ B cells. Isotype staining was used as a negative control for IL-10 detection in comparison to ctl (non-transplanted WT C57BL/6 mice), PBS-treated, or IL-21-treated LP/J mice. All shown experiments were conducted at least three times with *P < 0.05 and **P < 0.001 and *n* = 10/group

The observed T cell development triggered by IL-21 is expected to qualitatively change the content of secondary lymphoid organs due to enhanced RTE output (Fig. 1d, e). Although all spleens derived from LP/J transplanted animals looked similar in size to those from unirradiated control mice (Fig. 4a), higher GFP frequency (Fig. 4b) and counts of total as well as GFP^+^ splenocytes were detected in the IL-21 group (Fig. 4c). Not only did IL-21 administration culminate in full reconstitution of CD4^+^, CD8^+^, and CD19^+^ cells (Fig. 4d), but the majority of developed CD4^+^ and CD8^+^ T cells displayed a naïve phenotype (CD62L^hi^CD44^lo^) (Fig. 4e) with a normal TCRvβ distribution (Fig. 4f). To determine whether IL-21 administration to transplanted mice affects the nature of newly developed T lymphocytes, we next examined their functionality in response to TCR stimulation. No significant changes could be detected in their proliferative capacity (Additional file 1: Figure S2A) or ability to secrete a wide range of cytokines (Additional file 1: Figure S2B). Furthermore, all stimulated T cells expressed similar levels of granzyme B (Additional file 1: Figure S2C, D) with no depicted differences in their expression of *Bcl6*, *Nfat*, *T-bet*, *Gata3*, *Rorγt*, and *Foxp3* transcripts (Additional file 1: Figure S3). Together, our results suggest that IL-21 administration post-BMT stimulates thymopoiesis efficiently consequently triggering the emergence of peripheral naïve T cell pool displaying normal functionality.

### IL-21 drives regulatory B10 cell expansion in transplanted animals

IL-21 can exert actions on multiple lymphoid and myeloid populations with a potential role in the modulation/development of immune regulatory cells [63]. For instance, IL-21^−/−^ mice display high numbers of FOXP3-expressing Tregs, suggesting a function for IL-21 in blocking Treg development [28]. Elegant mechanistic studies further demonstrated that IL-21 impedes Treg generation and viability in vitro by inhibiting IL-2 production [64]. Besides Tregs, IL-21 supports B cell development in the BM, suggesting a possible parallel effect on regulatory B cells (B10) [38]. In support of this notion, activation and proliferation of B10 cells were reported to be dependent on both cognate interactions with CD4^+^ T cells and IL-21 during an inflammatory process [38]. Based on all these facts, we next scrutinized whether IL-21 administration affects the development of Tregs in mice undergoing de novo hematopoiesis and found a significant but trivial increase in CD4^+^CD25^+^FOXP3^+^ T cell number (Fig. 4g). However, the same IL-21 group displayed a pronounced increase in regulatory B cells based on the quantification of IL-10-competent CD1d^hi^CD5^+^ B cells (20% as opposed to 1–4% in control PBS-treated animals—Additional file 1: Figure S4; Fig. 4h). Therefore, IL-21 administration post-BMT enhances significantly the level of regulatory B10 cells along with a limited effect on Treg development.

### IL-21 protects transplanted mice from GVHD while preserving the GVT effect

**Fig. 5:**
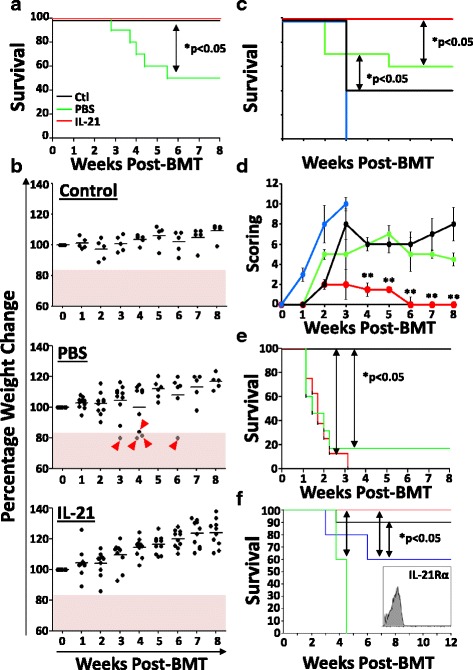
Effect of IL-21 on GVHD and GVT. **a** Survival curve following MHC-matched allogeneic BMT (C57BL/6 → LP/J) depicting control (syngeneic transplantation) in *black*, PBS-treated allogeneic BMT in *green*, and IL-21-treated allogeneic BMT in *red*. **b** Weight loss analysis of transplanted animals over 8 weeks post-BMT. *Pink area* represents a loss of ≥20%. Lost mice are indicated by *red arrowheads*. **c** Survival curve following MHC-matched allogeneic BMT depicting PBS-treated allogeneic BMT (WT C57BL/6 → LP/J) in *green*, IL-21-treated allogeneic BMT (WT C57BL/6 → LP/J) in *red*, PBS-treated allogeneic BMT (MμMT C57BL/6 → LP/J) in *blue*, and IL-21-treated allogeneic BMT (MμMT C57BL/6 → LP/J) in *black*. **d** Clinical score evaluation for the experiment shown in **c. e** Survival curve following complete MHC-mismatch allogeneic BMT (C57BL/6 → BALB/c). The color code for the groups is exactly the same one shown in **a. f** Survival curve for the GVT experiment conducted by challenging transplanted mice with P815 mastocytoma tumor cells. The color code for each group is as follows: *green* for BMT + PBS, *black* for BMT + IL-21, *blue* for BMT + DLI + PBS, and *red* for BMT + DLI + IL-21. **e** Representative flow cytometry analysis of IL-21Rα expression on the surface of the P815 tumor cell line. All shown experiments were conducted at least three times with *P < 0.05 and *n* = 10/group

So far, all BMT experiments were conducted using T cell-depleted BM to assess the thymus-boosting properties of IL-21 in the absence of GVHD-induced damages. To evaluate the effect of IL-21 on GVHD, we next supplemented the BM graft with mature RAG2p-GFP-derived allogeneic T cells (donor lymphocyte infusion (DLI)) and found that PBS-treated animals became ill as of the 3rd week post-BMT with a 50% survival rate (Fig. 5a) according to weight loss assessment (Fig. 5b). In sharp contrast, none of the IL-21-treated animals developed GVHD signs (Fig. 5a, b). As the proportion of regulatory B cells was enhanced in IL-21-treated animals (Fig. 4h), we next addressed whether these Bregs were behind the GVHD protection observed in transplanted LP/J mice. To test this hypothesis, T cell-depleted BM derived from MμMT mice (deficient in B cell development) was supplemented with mature T cells from RAG2p-GFP mice, then transplanted into LP/J animals followed by PBS or IL-21 treatments. Interestingly, 40% survival was obtained in the IL-21-treated group whereas all PBS control mice died by the 3rd week post-BMT (Fig. 5c, d). As these observations clearly indicate a role for Bregs in mediating GVHD protection, we next tested whether IL-21-enhanced Breg development would lead to similar outcome in a complete MHC-mismatch BMT model (C57BL/6 → BALB/c). Under this context, all BALB/c transplanted mice died within the 3rd week post-BMT with no observed differences between PBS and IL-21 groups (Fig. 5e).

GVHD induction is usually concomitant with GVT [65]. We therefore corroborated impaired GVT effect in IL-21-treated animals due to the absence of GVHD signs. To validate this hypothesis, LP/J recipient mice were transplanted with RAG2p-GFP-derived BM with or without DLI followed by a challenge the following day using the mastocytoma cell line P815 (DBA/2-derived). As predicted, all PBS-treated mice transplanted with T cell-depleted BM died around the 4th week (Fig. 5f; green line) whereas DLI led to 60% survival up to 12 weeks post-BMT (Fig. 5f; blue line). Surprisingly, 90% of challenged IL-21-treated mice transplanted with T cell-depleted BM survived (Fig. 5f; black line), whereas complete protection was obtained if animals underwent DLI (Fig. 5f; red line). It should be noted however that IL-21 can trigger apoptosis of IL-21R-expressing lymphomas [66, 67]. To confirm that the observed GVT effect is solely dependent on the anti-tumoral effect mediated by the newly developed thymus-selected T cells as opposed to a direct apoptosis-inducing effect mediated by IL-21 itself, we assessed the expression of the IL-21Rα chain on P815 by flow cytometry and found that it was indeed negative (Fig. 5f; histogram figure). We concluded therefore that IL-21 can bypass GVHD induction, at least in the MHC-matched allogeneic graft setting, while retaining beneficial GVT effects mediated by the newly generated T cells.

### IL-21 triggers T cell development in humanized mice

**Fig. 6:**
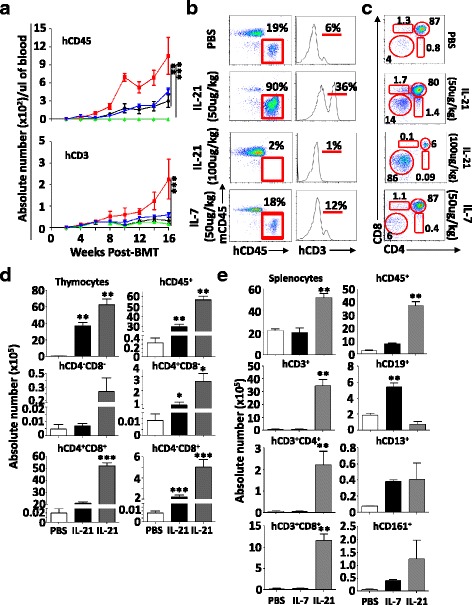
Human T cell development in NSG mice. **a** Peripheral blood analysis for the development of CD45^+^ and CD3^+^ human cells in NSG mice transplanted with T cell-depleted CB. The PBS group is shown in *black*, the IL-7 group at 50 μg/kg is shown in *blue*, the IL-21 group at 50 μg/kg is shown in *red*, and the IL-21 group at 100 μg/kg is shown in *green*. **b** Representative flow cytometry assessment of murine vs. human CD45^+^ cells (*left panels*) or human CD3^+^ T cells (*right panels*) in peripheral blood of NSG mice 16 weeks post-transplantation. **c** Representative flow cytometry analysis of thymic populations in transplanted NSG mice. **d** Absolute counts of all thymic subsets derived from the NSG experiment. **e** Absolute counts of all spleen subsets derived from the NSG experiment. The PBS group is shown in *white*, the IL-7 group at 50 μg/kg is shown in *black*, and the IL-21 at 50 μg/kg dose is shown in *gray*. All shown in vivo experiments were conducted three times with *P < 0.05, **P < 0.01, and ***P < 0.0001 and *n* = 10/group

To test whether human IL-21 displays a thymopoiesis-stimulating effect akin to its murine ortholog, we next transplanted immunodeficient NSG mice with T cell-depleted CB units. Two doses of human IL-21 were tested (50 and 100 μg/kg) versus IL-7 (50 μg/kg) or equal volume of PBS following the same injection schedule previously used for LP/J mice. Peripheral blood analysis over 16 weeks revealed a significant increase in both human CD45^+^ and CD3^+^ cells (Fig. 6a) only in the group receiving IL-21 at a dose of 50 μg/kg. The same IL-21-treated NSG group displayed 90% chimerism with over 30% circulating human CD3^+^ T cells (Fig. 6b). Besides the absence of a beneficial effect for IL-7, the high IL-21 dose (100 μg/kg) failed at improving human CD3^+^ T cell development (Fig. 6a), which could be explained by a blockade at the DN stage in the thymi of NSG transplanted mice (Fig. 6c). Detailed thymic analysis of the 50 μg/kg group showed pronounced human thymocyte counts (total and human CD45^+^ cells) with increased levels of DN, DP, and SP thymocytes (Fig. 6d). Their spleens contained higher counts of total and human CD45^+^ cells as well as significantly increased levels of CD3^+^, CD4^+^, CD8^+^, and CD19^+^ SP lymphocytes with no apparent differences in CD13^+^ (granulocytes) or CD161^+^ (NK/NKT) counts (Fig. 6e). Altogether, these observations clearly indicate that human IL-21 is equivalent to its murine ortholog in stimulating the development of MHC-restricted human CD3^+^ T cells in immunodeficient NSG mice.

## Discussion

**Fig. 7:**
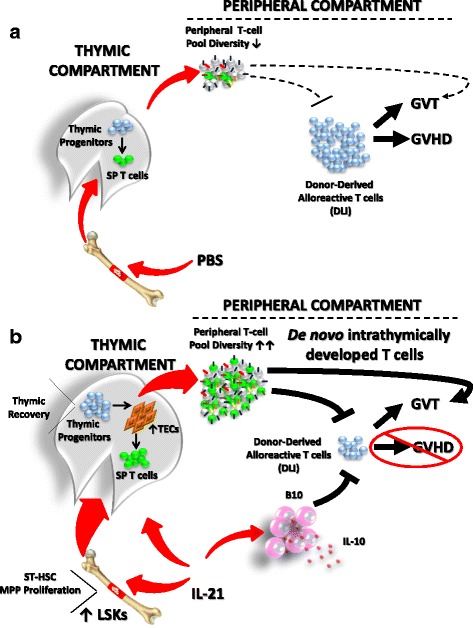
Graphical abstract. **a** BMT pre-conditioning damages the thymus, which results in poor thymopoiesis and the generation of a restricted peripheral TCR repertoire. Consequently, the GVT effect is limited along with the appearance of GVHD induced by DLI. **b** Following IL-21 administration to transplanted mice, ST-HSCs and MPPs expand most likely leading to an increase in the pool of thymus-seeding progenitors. In parallel, IL-21 triggers the proliferation of thymic progenitors, which positively affects TECs resulting in accelerated recovery of a naïve T cell pool with a diversified TCR repertoire. The newly generated thymus-derived T cells promote GVT, while GVHD is controlled by homeostatic pressure on allogeneic T cells and via the action of IL-21-induced B10 cells

The thymus is a specialized microenvironment dedicated to the generation of self-tolerant T cells with a broad TCR repertoire [68–70]. This is vital to ensure the development of adaptive immune responses without leading to autoimmune diseases. The importance of the thymus, however, must be reconciled with the fact that it is exquisitely sensitive to a range of external insults including those derived from the conditioning regimen used prior to BMT [71, 72]. Although pediatric patients can recover their thymic function post-transplantation, the latter remains blunted in aged individuals leading consequently to diverse clinical complications [6]. This explains why accelerated T cell recovery correlates with improved survival post-BMT [11–13, 16, 20, 24]. For de novo generation of thymus-derived naïve T cells to occur, three conditions must be fulfilled following BMT: (i) efficient HSC engraftment, (ii) active thymic seeding by BM-derived progenitor cells, and (iii) a thymic architecture conducive to progenitor migration/differentiation. Using a series of BMT experiments, we have shown that IL-21 administration to transplanted mice enhances de novo thymopoiesis by targeting both primary lymphoid tissues: the BM and the thymus (Fig. 7). More specifically, IL-21 promotes the expansion of ST-HSCs and MPPs within the BM-resident LSK population, which represents the source of building blocks required for rapid immune recovery [49, 51–55]. Although not directly assessed in our studies, we presume that such increase in the pool of BM progenitors enhances thymic seeding [60, 73]. In parallel, we demonstrated that IL-21 stimulates intrathymic T cell development directly by boosting the count of thymic progenitor cells while enhancing TEC recovery. The indirect effect of IL-21 on TECs is particularly important as these stromal cells guide the migration of BM-resident progenitors via the production of chemotactic gradients and mediate positive/negative selection of developing thymocytes [74]. Consistent with previous reports, our working model also stipulates the likely possibility that newly generated thymus-selected RTEs compete with peripheral donor-derived alloreactive T cells via a process we refer to as “homeostatic pressure” diminishing therefore the recipient’s susceptibility to GVHD while ensuring proper GVT effect [75, 76]. Our observations also hint to a possible role for IL-21 in inhibiting GVHD onset through the development, activation, and/or proliferation of B10 cells.

Thymopoiesis is a complex multilayered process with various factors at play [74]. Of all cytokines involved in T cell biology, IL-7 remains the central growth factor due to its role in the development, survival, and homeostatic proliferation of T cells [48]. Based on these properties, it was extensively studied in the context of BMT where it was shown to exert powerful thymopoietic-stimulating effects [77–89]. By directly comparing the pharmacological potential of IL-7 and IL-21 using the optimal IL-21 dose (50 μg/kg) previously identified, we found no significant increase in RTE output triggered by IL-7 administration to LP/J recipient mice (Fig. 1b–d). This is not surprising as IL-7 supports thymopoiesis when administered at doses ranging from 250 to 500 μg/kg (e.g., five- to tenfold higher than the dose used in our BMT experiments) [79, 84, 87]. It must be noted, however, that IL-21 cannot support the development of SP thymocytes in the absence of IL-7 signaling (Fig. 1g). This is consistent with our previous in vitro observations where IL-21 was found to be inert at driving T cell differentiation due to its inability to trigger ERK phosphorylation in TCR-engaged DP thymocytes [35]. Thus, T cell reconstitution could be enhanced/accelerated by IL-21 via the induction of BM/thymic progenitor expansion but relies exclusively on IL-7 signaling to exert its pharmacological effect.

One of the most seminal observations made in the herein study was the absence of GVHD signs in IL-21-treated animals while the GVT effect was retained. This is counterintuitive as GVT is usually associated with GVHD induction [65]. Furthermore, the IL-21/IL-21R signaling axis was previously reported to exacerbate GVHD in a CD4-dependent manner involving B cell expansion and production of autoantibodies [73, 90, 91]. Our study puts at play two important factors, which may help depict the discrepancy between our observations and those reported by others. First, GVHD and GVT are usually mediated by allogeneic T cells (e.g., DLI) transferred along or after BMT [65]. The purpose of DLI is to provide transient protection against infectious disease while ensuring eradication of post-conditioning regimen-resistant cancer cells [59]. As T cell development is severely delayed following allogeneic BMT, donor-derived alloreactive T cells can expand in lymphopenic recipients consequently increasing recipients’ susceptibility to GVHD [75, 76, 92–94]. Under these circumstances, the endogenous production of any pro-inflammatory cytokine(s), including IL-21, during GVHD onset would exacerbate the magnitude of the disease [75, 76, 92, 93]. In this regard, the accelerated appearance of thymus-selected RTEs triggered by exogenous IL-21 administration would not only compete with peripheral alloreactive T cells to access secondary lymphoid organs, homeostatic cytokines, and self-MHC ligands impeding therefore alloreactive T cell expansion/activation, but it will also ensure efficient immune surveillance and tumor rejection as we have observed with the P815 challenge experiment conducted in IL-21-treated animals (Fig. 6). Second, GVHD could be alleviated through the concrete action of B10 cells, which were previously reported to be induced by IL-21 [38]. Interestingly, B10 cells are potent regulators of macrophages and dendritic cell function, which suggest that IL-21-induced B10 cells may contribute to GVHD inhibition by restraining monocyte and dendritic cell-mediated stimulation of alloreactive T cells [95–99]. Although we did not directly investigate the Breg mechanism of action in our BMT models, the decreased survival rate observed in IL-21-treated LP/J mice transplanted with B cell-deficient BM supports a central role for B10 cells in alleviating GVHD (Fig. 5c, d). This prompts us to formulate the hypothesis that IL-21-mediated induction of B10 cells would be beneficial for controlling GVHD in a complete MHC-mismatch model (C57BL/6 → BALB/c). Since the complete loss of all transplanted IL-21-treated BALB/c animals occurred within the first 3 weeks post-BMT, one can argue that the strong alloreactive response mediated by allogeneic T cells overrides the B10 suppressive effect. Furthermore, potent production of interferon-gamma triggered by strong alloreactivity may counterbalance B10 cell expansion in vivo [38]. Besides, the observed alloreactivity in the complete MHC-mismatch BMT model occurred faster (less than 3 weeks) than the time required for de novo B (~3–4 weeks) and T cell development (~5 weeks) previously observed in LP/J recipient mice. As for the discrepancy between our observations and the exacerbated xeno-GVHD onset observed by Wu et al. in NSG mice, two central points must be discussed: the source of DLI and the bioavailability of human IL-21. In the study by Wu et al., peripheral blood mononuclear cells containing mature T cells were infused instead of T cell-depleted CB. In this context, mature B and T cells would certainly promote a superior and faster xenoreactivity in NSG mice leading consequently to xeno-GVHD. Second, human IL-21 was undetectable in peripheral blood of hydrodynamically transfected NSG mice beyond 5 days. As we administered human IL-21 over 2 weeks, we presume based on our LP/J observations that our approach ensures higher de novo T cell development with the potential induction of IL-21-induced human B10 cells, thus significantly improving NSG survival. Although evidence suggests that both positive and negative selection can occur in NSG mice [40, 100], we cannot preclude the possibility of xeno-GVHD development in NSG mice beyond 16 weeks post-transplantation. Additional mechanistic studies are therefore warranted to further demystify IL-21’s mode of action in NSG mice.

## Conclusions

In summary, our study clearly establishes a role for IL-21 in immune recovery following BMT, where it acts as a regulator of BM progenitor expansion as well as on the earliest phases of intrathymic T cell development and recovery of the stromal compartment. Its ability to simultaneously affect the BM and thymic compartments while repressing alloreactive T cells gives it an advantageous edge over currently tested therapies. Although our studies suggest that IL-21 could be exploited as an immunotherapeutic agent for the restoration of essential immune system functioning following ablative therapy, its impact on inducing/promoting autoimmune diseases in BMT patients remains elusive. Therefore, future pre-clinical and clinical trials should foresee the use of human IL-21 to determine its safety and efficacy in facilitating immune recovery post-BMT.

## Additional file

Additional file 1: Figure S1. Gating strategies for LSKs. BM cells collected from WT or IL-21R^−/−^ C57BL/6 mice were treated in vitro for proliferation, then stained with the Lin^−^Sca1^+^c-kit^+^ antibody cocktail prior to Ki-67^+^ incorporation analysis. All described experiments were conducted at least three times with *n* = 5/group. **Figure S2.** Functional characterization of T cells. (A) Representative cell trace dilution analysis on CD4^+^ or CD8^+^ T cells derived from ctl (unirradiated), PBS-treated, or IL-21-treated LP/J recipient mice. (B) Cytokine quantification by ELISA from T cells derived from the same groups described in panel (A). (C) Representative flow cytometry analysis of granzyme B expression. (D) Quantification of T cells expressing granzyme B. For all presented studies, T cells were stimulated with CD3-CD28 dynabeads for 48 h prior to analyses. All described experiments were conducted at least three times with an *n* = 5/group. **Figure S3.** Molecular characterization of T cells. T cells sorted from ctl, PBS-treated, or IL-21-treated LP/J recipient mice were analyzed for their expression of various transcription factors involved in T cell differentiation. All described experiments were conducted at least three times with *n* = 5/group. **Figure S4.** Gating strategies for Breg analysis. For detection of IL-10-producing Bregs, CD19^+^ B cells were first isolated from spleens of treated mice (isotype shown by the filled gray histogram), then stained after in vitro treatment with CD1d and CD5 antibodies. The B cell subset CD1dhiCD5^+^ was gated prior to IL-10 assessment by intracellular staining. All described experiments were conducted at least three times with *n* = 5/group. (PDF 2111 kb)

## Abbreviations

B10: IL-10 producing regulatory B cells
BM: Bone marrow
BMT: Bone marrow transplantation
CB: Cord blood
DLI: Donor lymphocyte infusion
DN: Double-negative
DP: Double-positive
GVHD: Graft-versus-host-disease
GVT: Graft-versus-tumor
HSC: Hematopoietic stem cells
IL: Interleukin
IL-21R: IL-21 receptor
LSK: Lin^−^Sca1^+^c-kit^+^
LT-HSCs: Long-term hematopoietic stem cells
MHC: Major histocompatibility complex
MPP: Multipotent progenitors
RTE: Recent thymic emigrant
SP: Single-positive
ST-HSCs: Short-term hematopoietic stem cells
TCR: T cell receptor
TEC: Thymic epithelial cell
Tregs: Regulatory T cells
WT: Wild-type
