# Supplementary material for: Interleukin-21 promotes thymopoiesis recovery following hematopoietic stem cell transplantation
Source: J Hematol Oncol. 2017 Jun 14;10:120. doi: 10.1186/s13045-017-0490-3 (PMC5471903; doi:10.1186/s13045-017-0490-3)
Supplement: Additional file 1: Figure S1. — Gating strategies for LSKs. BM cells collected from WT or IL-21R−/− C57BL/6 mice were treated in vitro for proliferation, then stained with the Lin−Sca1+c-kit+ antibody cocktail prior to Ki-67+ incorporation analysis. All described experiments were conducted at least three times with n = 5/group. Figure S2. Functional characterization of T cells. (A) Representative cell trace dilution analysis on CD4+ or CD8+ T cells derived from ctl (unirradiated), PBS-treated, or IL-21-treated LP/J recipient mice. (B) Cytokine quantification by ELISA from T cells derived from the same groups described in panel (A). (C) Representative flow cytometry analysis of granzyme B expression. (D) Quantification of T cells expressing granzyme B. For all presented studies, T cells were stimulated with CD3-CD28 dynabeads for 48 h prior to analyses. All described experiments were conducted at least three times with an n = 5/group. Figure S3. Molecular characterization of T cells. T cells sorted from ctl, PBS-treated, or IL-21-treated LP/J recipient mice were analyzed for their expression of various transcription factors involved in T cell differentiation. All described experiments were conducted at least three times with n = 5/group. Figure S4. Gating strategies for Breg analysis. For detection of IL-10-producing Bregs, CD19+ B cells were first isolated from spleens of treated mice (isotype shown by the filled gray histogram), then stained after in vitro treatment with CD1d and CD5 antibodies. The B cell subset CD1dhiCD5+ was gated prior to IL-10 assessment by intracellular staining. All described experiments were conducted at least three times with n = 5/group. (PDF 2111 kb) [file 13045_2017_490_MOESM1_ESM.pdf]

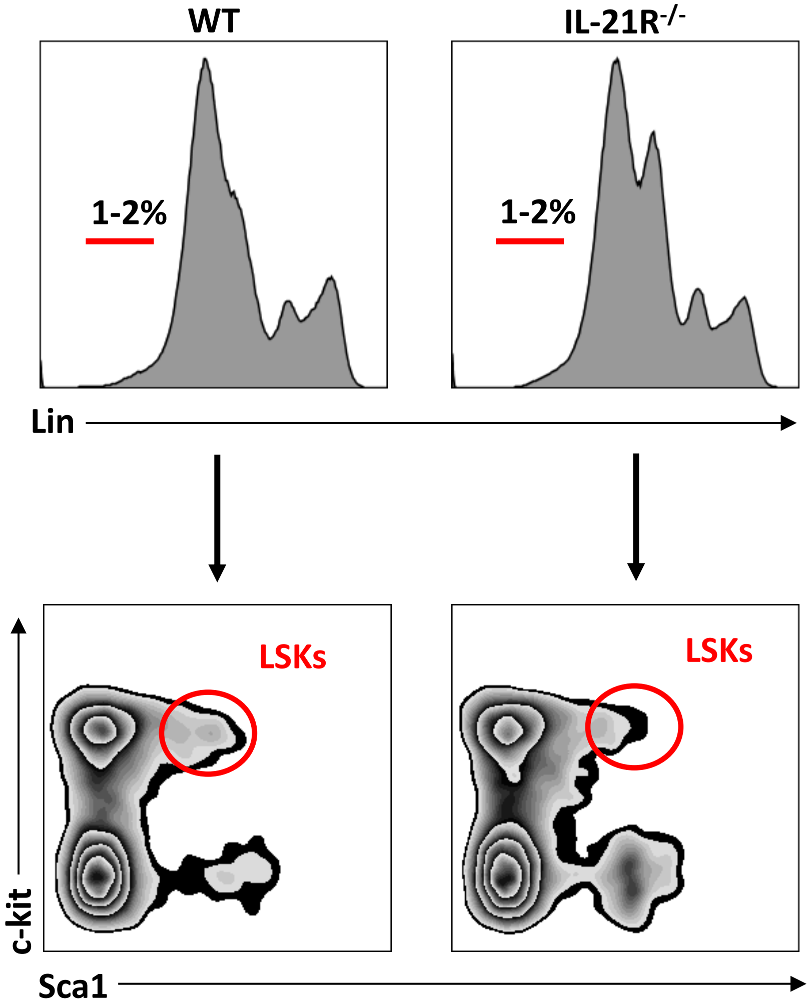

Figure S1: Gating strategies for LSKs. BM cells collected from WT or IL-21R$^{-/-}$ C57BL/6 mice were treated *in vitro* for proliferation then stained with the Lin^{-}Sca1^{+}c-kit^{+} antibody cocktail prior to Ki-67^{+} incorporation analysis. All described experiments were conducted at least three times with n=5/goup.

FIGURE S1

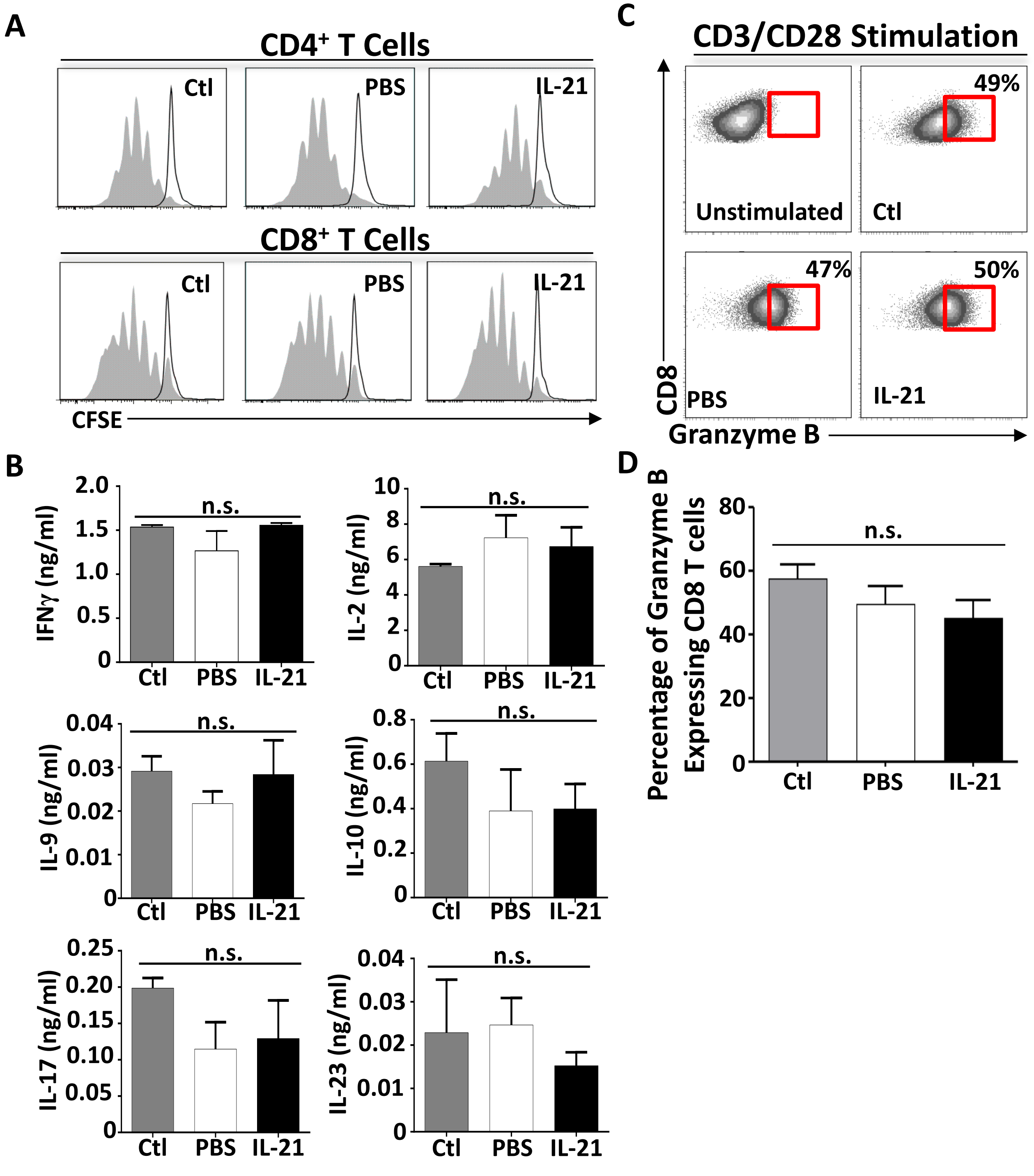

Figure S2: Functional characterization of T cells. (**A**) Representative cell trace dilution analysis on CD4+ or CD8+ T cells derived from ctl (unirradiated), PBS- or IL-21-treated LP/J recipient mice. (**B**) Cytokine quantification by ELISA from T cells derived from the same groups described in panel (**A**). (**C**) Representative flow-cytometry analysis of Granzyme B expression. (**D**) Quantification of T cells expressing granzyme B. For all presented studies, T cells were stimulated with CD3-CD28 dynabeads for 48 hrs prior to analyses. All described experiments were conducted at least three times with an n=5/group.

FIGURE S2

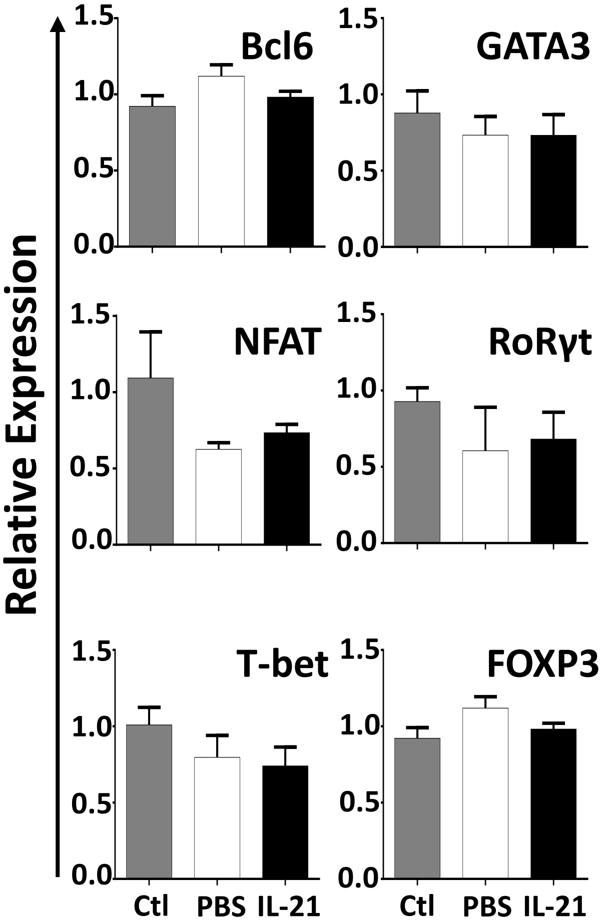

Figure S3: Molecular characterization of T cells. T cells sorted from ctl, PBS- or IL-21-treated LP/J recipient mice were analyzed for their expression of various transcription factors involved in T-cell differentiation. All described experiments were conducted at least three times with n=5/goup.

FIGURE S3

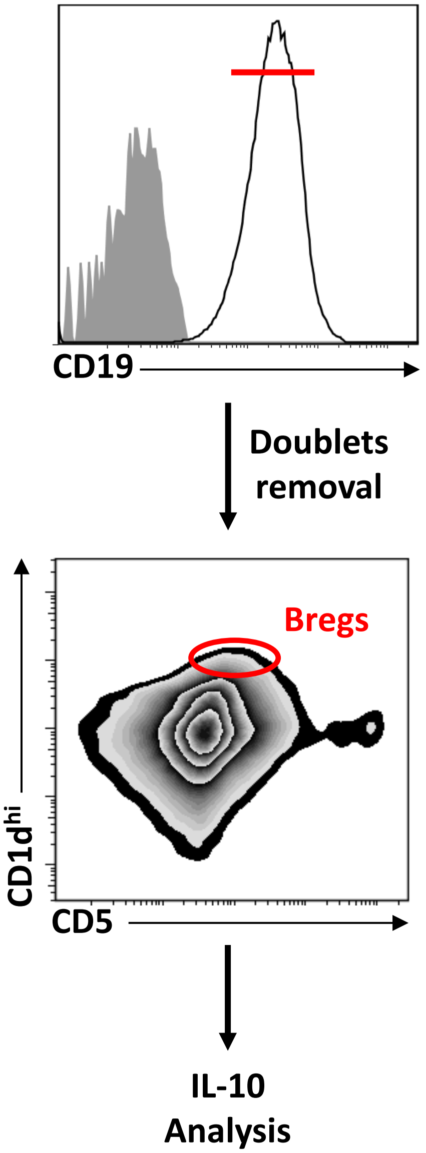

Figure S4: Gating strategies for Bregs analysis. For detection of IL-10-producing Bregs, $CD19^+$ B cells were first isolated from spleens of treated mice (isotype shown by the filled grey histogram) then stained after *in vitro* treatment with CD1d and CD5 antibodies. The B-cell subset $CD1d^{hi}CD5^+$ was gated prior to IL-10 assessment by intracellular staining. All described experiments were conducted at least three times with n=5/goup.

FIGURE S4
